# Laparoscopic Heller's cardiomyotomy in cirrhosis with oesophageal varices

**DOI:** 10.4103/0972-9941.65164

**Published:** 2010

**Authors:** Abhay N Dalvi, Pinky M Thapar, Nitin M Narawane, Rippan N Shukla

**Affiliations:** Department of Minimal Invasive Surgery, Jupiter Hospital, Thane, Maharashtra, India; 1 Department of Gastroenterology, Jupiter Hospital, Thane, Maharashtra, India

**Keywords:** Achalasia, cirrhosis, esophageal varices, laparoscopic cardiomyotomy

## Abstract

Surgical intervention in cirrhosis of liver with portal hypertension is associated with increased morbidity and mortality. This is attributed to liver decompensation, intra-operative bleeding, prolonged operative time, wound related and anaesthesia complications. Laparoscopic surgery in cirrhosis is advantageous but is associated with technical challenges. We report one such case of hepatitis C cirrhosis with oesophageal varices and symptomatic achalasia cardia, who was successfully treated by laparoscopic cardiomyotomy after thorough preoperative workup and planning. In the review of literature on pubmed, no such case is reported.

## INTRODUCTION

Cirrhosis of liver with portal hypertension is associated with high risk for any surgical intervention.[1] Laparoscopic surgery offers advantages of decreased tissue trauma and surgical stress in cirrhosis.[2] Combination of Hepatitis C cirrhosis with oesophageal varices and symptomatic achalasia cardia is rare. Laparoscopic cardiomyotomy is a well-established technique to treat achalasia. Surgery involves separation of oesophageal and gastric musculature from underlying mucosa to release the high pressure zone of lower oesophageal sphincter (LES)[3] However, dissection in the submucosal plane in the presence of varices is technically challenging. Bleeding obscuring the vision is one of the obstacles of this procedure that can lead to complication of oesophageal mucosal perforation. Thorough pre-operative investigations, planning, meticulous dissection are required to tackle this problem by a laparoscopic approach. PUBMED search shows no reported case of laparoscopic cardiomyotomy in patient of cirrhosis with oesophageal varices.

## CASE REPORT

A 53-year-old lady was referred to us for surgical management of achalasia cardia. In the past, she had sustained severe gastroenteritis (30 years ago) for which she was transfused 2 units of blood. She developed jaundice 25 years ago which responded to medical management. Since 6 years, she had progressive dysphagia to liquids and solids associated with weight loss and nocturnal cough. She underwent oesophageal manometry and upper GI endoscopy 2 years ago and was diagnosed to have achalasia cardia. Apart from classical findings of dilated oesophagus and narrowing at the gastro-oesophageal (GE) junction, endoscopy revealed the presence of three columns of grade I-II varices. She had no history of GI bleed. During the same time, she was also detected to have hepatitis C with high viral load and was treated with interferon decreasing her viral load.

She received botulinum toxin injection for treatment of achalasia cardia, but had recurrence of symptoms of dysphagia within few months of the injection. Nocturnal dry cough progressed to a distressing level and fear of endoscopic dilatation inducing variceal bleed, led for a referral for a surgical option.

On examination, she had mild hepatomegaly, but no evidence of splenomegaly or ascitis.

**Figure 1:**
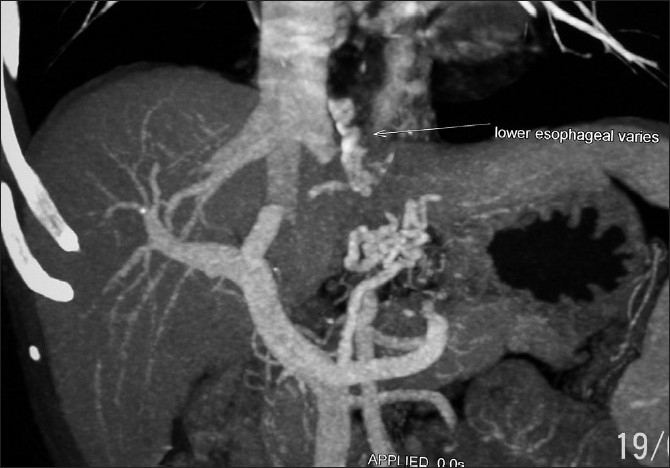
CT angiogram showing collaterals marked with arrows.

Repeat endoscopy revealed grade II oesophageal varix on the right wall, grade I column to the left of midline and grade II on posterior wall. A CT angiography was done, which showed dilated left gastric vessel, collaterals around the oesophago-gastric junction and lower oesophageal varices [Figure 1]. Her Liver function tests were normal and she was categorized as child A cirrhosis.

**Figure 2:**
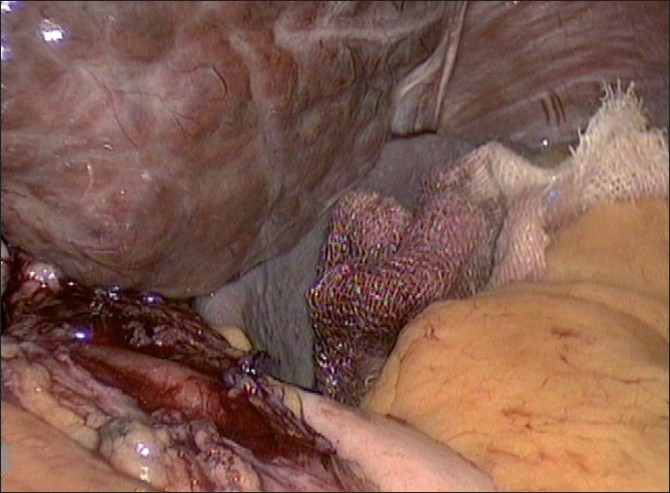
Completed cadiomyotomy showing cirrhotic liver, cardiomyotomy and endoscopic illumination.

**Figure 3:**
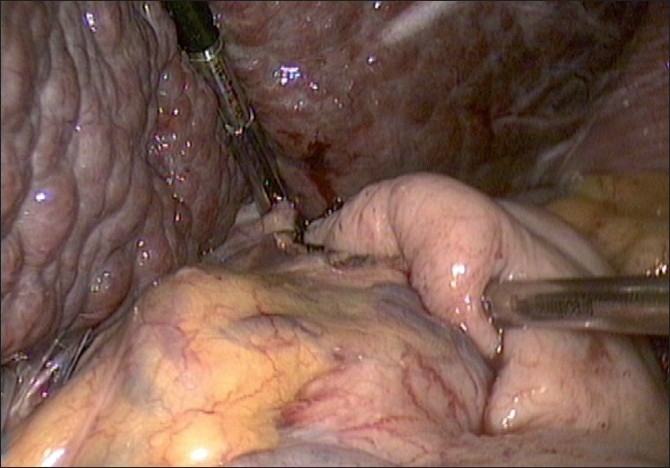
Completed fundoplication showing dilated collaterals and liver retraction.

Laparoscopic attempt at cardiomyotomy was planned. Standard ports for laparoscopic cardiomyotomy were made. Findings included an enlarged cirrhotic liver, large left gastric vessels and collaterals in the left gastro-hepatic ligament. Left lobe was retracted using a 5 mm instrument grasping the right crus. Staying in midline, anterior myotomy was done using harmonic shear and extended for 7 cm, with 1 cm extension over anterior wall of stomach [Figure 2]. Thickening of oesophageal wall with fibrosis was encountered. There was the presence of sub-mucous varix, which was controlled with harmonic shear. Intra-operative endoscopy was done to confirm the adequacy of myotomy. After satisfactory findings, anterior Dor fundoplication was done [Figure 3]. The procedure was completed in 80 min with minimum blood loss of 50 ml. She had good post-operative recovery.

Conray gram done on day 1 documented free flow of dye across the GE junction. Her nocturnal cough and dysphagia disappeared since the day of surgery. She was on primary prophylaxis, tab propanolol to prevent bleeding from varices. At 18 months' follow up, patient was on full diet with no complaints.

## DISCUSSION

Cirrhosis of liver with documented varices on endoscopy involves development of dilatation of submucosal and paraoesophageal venous channels specially in the lower oesophagus and upper stomach. Surgery on or around these regions in patients with varices is challenging, and prone to bleeding. Achalasia cardia is a primary oesophageal motility disorder characterized by failure of relaxation of hypertensive LES on swallowing and aperistalsis of the body of the oesophagus.[4,5] Progressive dysphagia more to liquids than solids, regurgitation, retrosternal burning, weight loss are the common symptoms. Nocturnal coughing with pulmonary infiltrates that was one of the presenting complaint in our patient is rare but indicates severe disorder.[6]

Modification in diet and lifestyle, pharmacotherapy,[7] botulinum toxin injection[8] balloon dilatation[9,10] and surgery[11] are the treatment options available in patients with achalasia cardia. Botulinum toxin injection was tried in our case as patient was high risk for surgery due to varices. It acts by inhibiting calcium-dependent release of acetylcholine from nerve terminals, but 50% patients develop recurrence within 6 months,[11,12] While nocturnal cough never improved, symptoms of dysphagia had recurred in a short time. Balloon dilatation is an established therapy involving forceful stretching and disruption of LES, but was deferred due to the presence of varices. Fear of developing pulmonary infiltrates due to persistent and increasing nocturnal cough, recurrence of symptoms of dysphagia after conservative treatment modalities led to a surgical referral.

Cardiomyotomy with or without an anti-reflux procedure is well established and effective modality for treatment of achalasia cardia. Advantages of decreased pain, in-hospital stay, less tissue trauma and early recovery has given laparoscopic approach an absolute advantage over open surgery.[13]

In our patient, there was this unreported problem of associated cirrhosis and oesophageal varices, and advice of laparoscopic or open approach had to be guarded. The surgery had to be meticulously planned and knowledge of venous channels in and around the lower oesophagus and upper stomach was absolutely mandatory. This was managed by CT angiography and endoscopy. Co-ordination between the endoscopist, radiologist and the surgical team allowed us to proceed with the laparoscopic approach. Further, recent reports documenting feasibility of laparoscopic surgery in cirrhotics for bleeding varices with emphasis on technological advancements and surgical expertise[14] encouraged us to push for a laparoscopic over open approach with background of 22 laparoscopic cardiomyotomies in non-cirrhotics.

Risk of bleeding due to the presence of varices in the plane of dissection, dilated para-oesophagogastric collaterals, manipulation of non-pliable nodular left liver lobe, fibrosis due to botulinum toxin injection were the technical challenges. Discussion and review of the investigations had revealed a relatively "risk free" window at 12 O Clock position where the dissection had to be done to keep bleeding to minimum and tackle the 1 O'clock submucosal varix with caution. The same was achieved with use of harmonic scalpel. Submucosal fibrosis and scarring due to history of botulinum toxin injection is known[12] and was encountered in the present case. Intraoperative endoscopy helped to confirm accurate completion of myotomy across the GE junction.

Post-operative instant relief from nocturnal cough and dysphagia is the aim of cardiomyotomy in patients with achalasia cardia, and the same was achieved using the laparoscopic technique with uneventful post-operative recovery.

The literature search on Medline (Pubmed) does not reveal any case of laparoscopic myotomy for achalasia cardia in patient of oesophageal varices.

## CONCLUSION

Technical challenges in laparoscopic surgery like cirrhosis can be overcome with thorough preoperative planning, precise and meticulous dissection.
